# Primary alveolar soft part sarcoma of uterine corpus: a case report with immunohistochemical, ultrastructural study and review of literature

**DOI:** 10.1186/s12957-016-0780-1

**Published:** 2016-02-02

**Authors:** Giovanna Giordano, Tiziana D’Adda, Elena Varotti, Giuseppe Crovini, Enrico Maria Silini

**Affiliations:** 1 Department of Biomedical, Biotechnological and Translational Sciences, Pathological Anatomy and Histology Unit, Faculty of Medicine, University of Parma, Via Antonio Gramsci, 14, 43126 Parma, Italy; 2 Department of Gynaecology and Obstetrics, Hospital of Fidenza, Parma, Italy

**Keywords:** Alveolar soft part sarcoma, Chromosomal translocation, TFE3 fusion protein

## Abstract

**Background:**

Alveolar soft part sarcoma (ASPS) is a rare mesenchymal malignancy. ASPS usually occurs most commonly in the deep soft tissues of the thigh and buttock or the head and neck regions.

ASPS that originate from the uterine corpus are even more rare, with only 10 previous cases reported in the English literature.

**Case presentation:**

In our case, the alveolar features were completely lost and the tumour shows a solid, non-alveolar pattern and the nuclei have marked variation in nuclear size, and multinucleation. The correct pathological diagnosis has been made by immuno- histochemical and ultrastructural features, which rvealed overexpression of TFE3 and peculiar cytoplasmic crystalline inclusions.

In this paper, an additional case of primary ASPS of uterine corpus is reported with immunohistochemical, ultrastructural study and review of literature in the effort to delineate its clinical and pathological features. In this unusual site, the diagnosis can be problematic because ASPS can mimic other primary or metastatic uterine neoplasms.

**Conclusions:**

Thus, in this unusual presentation an essential diagnostic marker is the nuclear over-expression of TFE3 as well as ultrastructural study, which reveals the presence of peculiar cytoplasmic crystalline inclusions.

## Background

Alveolar soft part sarcoma (ASPS) is a rare mesenchymal malignancy with distinctive histologic and ultrastructural appearance. ASPS was first described in 1952 by Christopherson et al. as a neoplasm with of uncertain histogenesis whose cells were arranged in a pattern mimicking the small air sacks (alveoli) of the lung [1].

In fact, this neoplasm usually shows uniform, organoid nests of polygonal cells, separated by fibrovascular septa and delicate capillary-sized vascular channels. A prominent cellular dyscohesion within the nests results in a distinctive pseudo-alveolar pattern. Sometimes, the alveolar features can be completely lost and the tumour may show a solid, “non-alveolar” pattern [2]. Usually, the nuclei are round-to-polygonal and vesicular, with prominent nucleoli, but cells with marked variation in nuclear size, nuclear-cytoplasmatic inclusions, and multinucleation have been reported [3–5]. The cytoplasms are abundant granular and may contain periodic acid-Schiff-(PAS) positive, diastase (D)-resistant crystalline structures, rhomboid or rod-like in shape [6]. The first ultrastructural analysis of ASPS, made by Shipkey et al. in 1964 [6] and following studies confirmed the presence of distinctive cytoplasmic crystals which typically were intermingled with dense granules [7–9]. More recently, Ladanyi et al., by a combined ultrastructural and immunohistochemistry study, have demonstrated that these crystals consist of aggregates of the monocarboxylate transporter protein MCT1 and its cellular chaperone CD147 [10].

Recent cytogenetic studies revealed that ASPSs are characterized by specific chromosomal translocation der(17)t(X;17) (p11;q25) that fuses the transcription factor 3 (TFE3) gene at Xp11 to the ASPL gene at 17q25, producing an ASPL–TFE3 fusion protein [11]. This results in the aberrant and strong nuclear expression of TFE3 which is seen almost exclusively in tumours harbouring the TFE3 gene fusions, such as ASPSs and rare paediatric renal carcinomas [12].

ASPS usually affects adolescents and young adults in the second and third decades with slight female predominance [3]. In adults, this malignancy occurs most commonly in the deep soft tissues of the thigh or buttock, while in children and infants, the head and neck regions are often involved [3]. However, many reports have demonstrated that ASPS can be observed in unusual sites such as the mediastinum, stomach, breast, bone, and urinary bladder [13–19].

This neoplasm has been reported also in the female genital tract [20–26]. As far as we are aware, only 10 cases of ASPS of uterine corpus have been previously reported [4, 5, 27–32]. In these unusual sites, the diagnosis can be problematic because ASPS can mimic other primary or metastatic neoplasms.

In this paper, an additional case of primary ASPS of uterine corpus is reported with immunohistochemical, ultrastructural study and review of literature in the effort to delineate its clinical and pathological features.

## Case presentation

A 66-year-old female was hospitalized for atypical vaginal bleeding and anaemia. On gynecologic examination, the uterus was enlarged and the cervix was prolapsed. Transvaginal ultrasound identified a well-circumscribed, intramural nodule measuring 5 cm in diameter, located in the uterine corpus. The patient underwent total abdominal hysterectomy and bilateral salpingo-oophorectomy. Intraoperatively, no ascites or adhesions were seen surrounding the uterus, ovaries, and salpinges. No enlarged pelvic lymph nodes were noted neither peritoneal lesions were found.

**Fig. 1:**
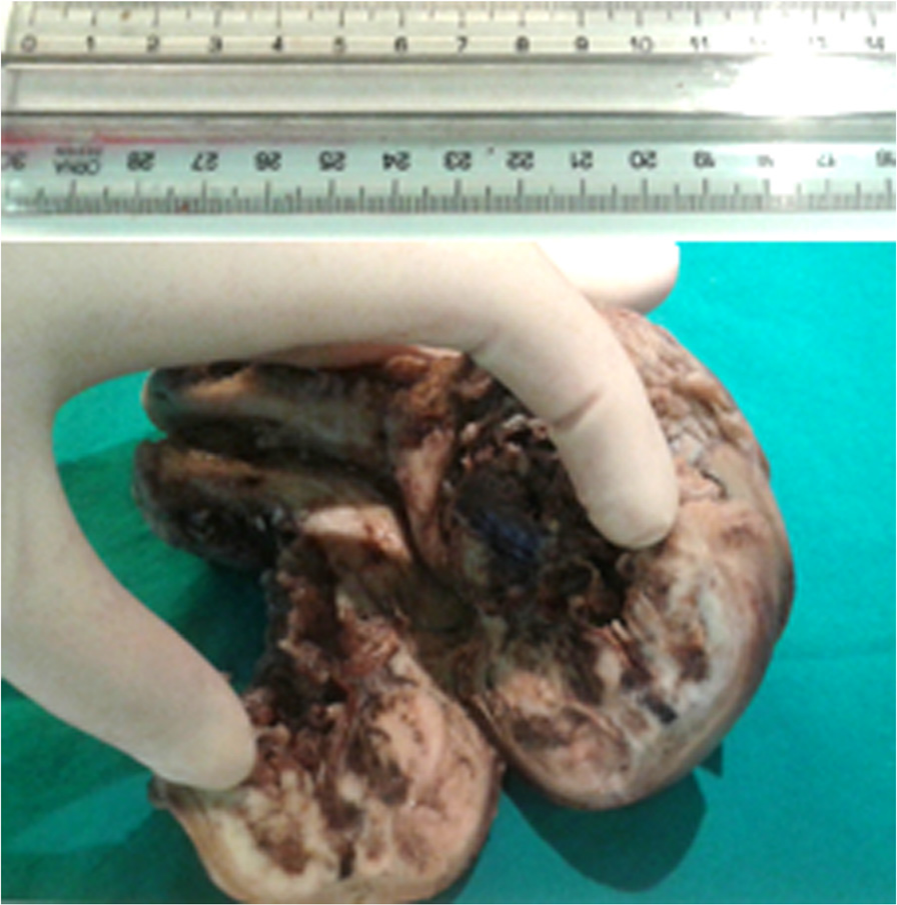
On cut sections, the tumour was well circumscribed, soft, with irregular border and showed areas which varied from yellow, brownish and grey, with a central haemorrhagic area

Gross pathologic examination revealed thickening of the cervical mucosa and an intramural, sharply circumscribed, round, firm, grey-white nodule with trabeculated cut surface located in the endocervix. The uterine corpus was enlarged and deformed because of an intramural nodule, measuring 5 cm in diameter. On cut section, this lesion was well circumscribed, soft, with irregular border, colour varying from yellow, brownish and grey and a large haemorrhagic zone centrally located (Fig. 1). The ovaries and salpinges were macroscopically unremarkable.

**Fig. 2:**
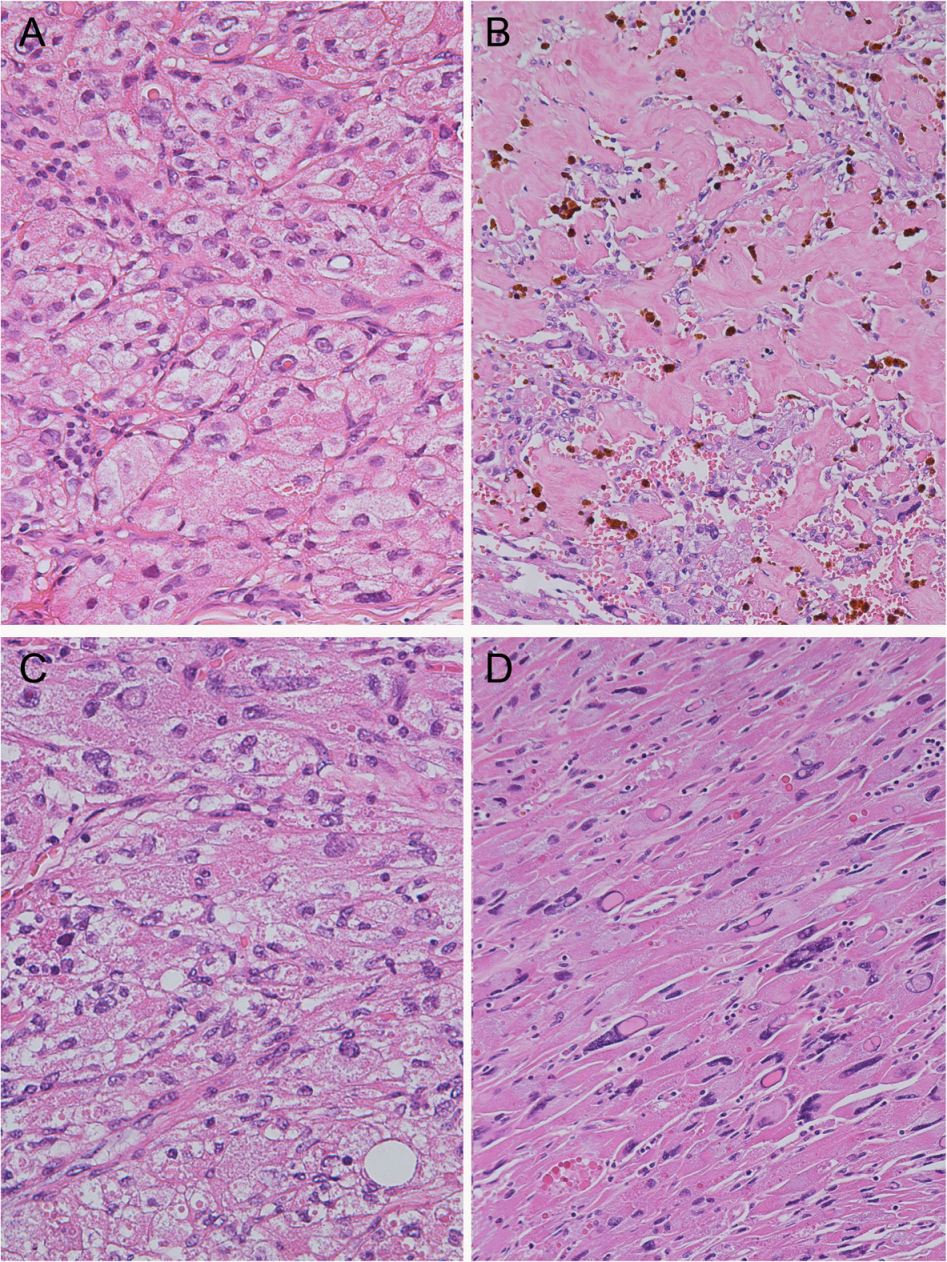
Histologically, the tumour showed nests of neoplastic cells with abundant eosinophilic granular cytoplasm, distinct borders and vesicular nuclei with prominent nucleoli (**a** haematoxylin and eosin stain ×400). Extensive degenerative changes, such as hyalinization with haemorrhagic and hemosiderin deposits, were observed (**b** haematoxylin and eosin stain ×200). Other cells were large with clear vacuolated cytoplasms (**c** haematoxylin and eosin stain ×400). In some areas, the organoid pattern was lost and the tumour showed solid growth, made spindle cells with nuclear, pleomorphism, hyperchromasia, pseudo-inclusions and multinucleations (**d** haematoxylin and eosin stain ×200)

Histologically, the cervix showed atrophic epithelium and an intramural leiomyoma. The nodule of the uterine corpus was characterized by neoplastic cells arranged in an organoid pattern (Fig. 2a), with nests surrounded by a delicate fibrovascular septa highlighted by PASD and anti-CD34 stains. Extensive degenerative changes, such as hyalinization, haemorrhage and hemosiderin deposits were observed (Fig. 2b). Most cells had abundant eosinophilic granular cytoplasm, distinct border and vesicular nuclei with prominent nucleolus (Fig. 2a). Other cells were large, with clear vacuolated cytoplasms (Fig. 2c). In some areas, the organoid pattern was lost and the tumour showed solid growth; in these areas, the cells were more spindled and showed nuclear pleomorphism, hyperchromasia and pseudo-inclusions and multinucleation (Fig. 2d). Necrosis and mitoses were absent.

**Fig. 3:**
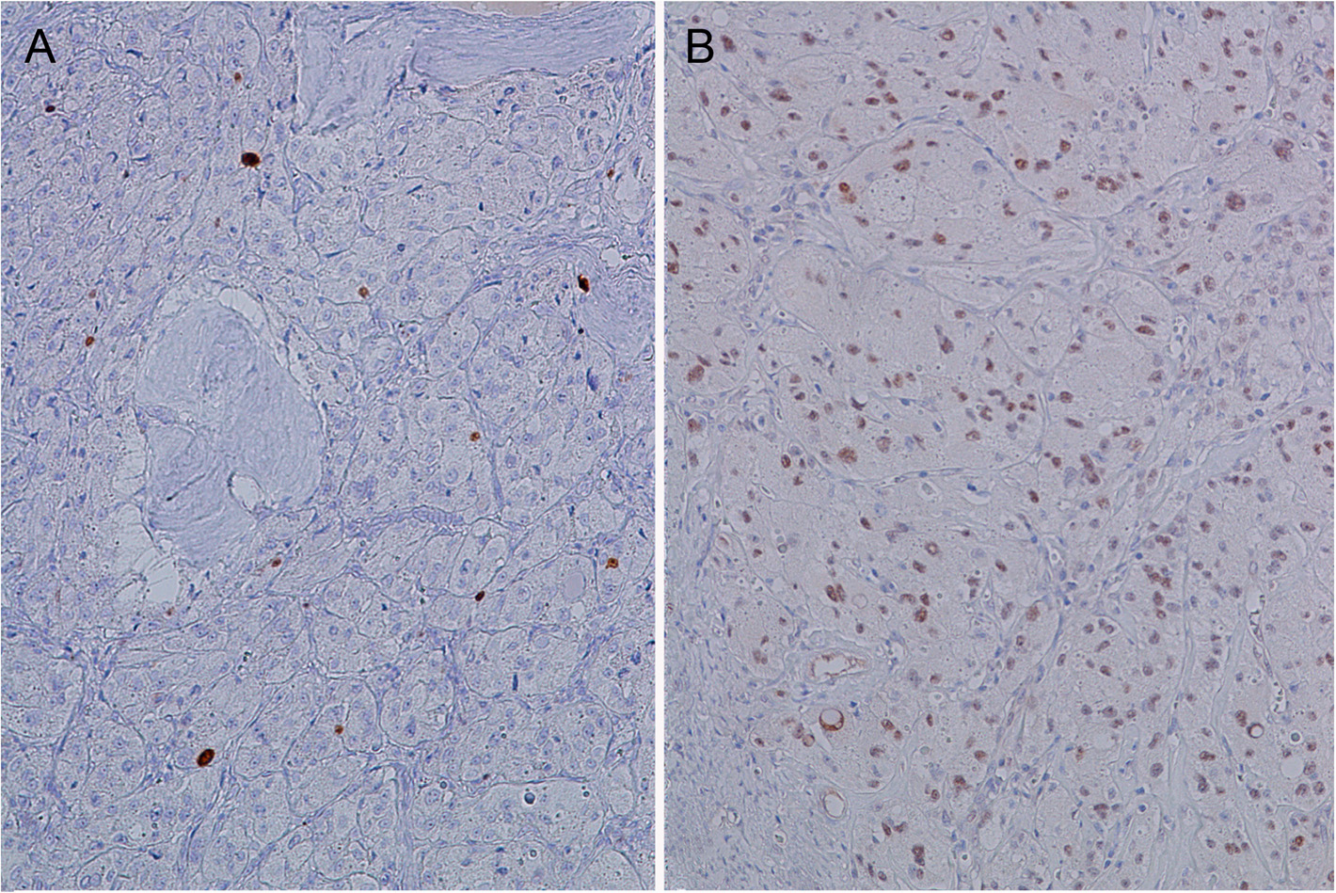
Ki 67 index was very low (**a** ×200). Strong nuclear positivity to TFE3 was observed in all cells (**b** ×200)

**Fig. 4:**
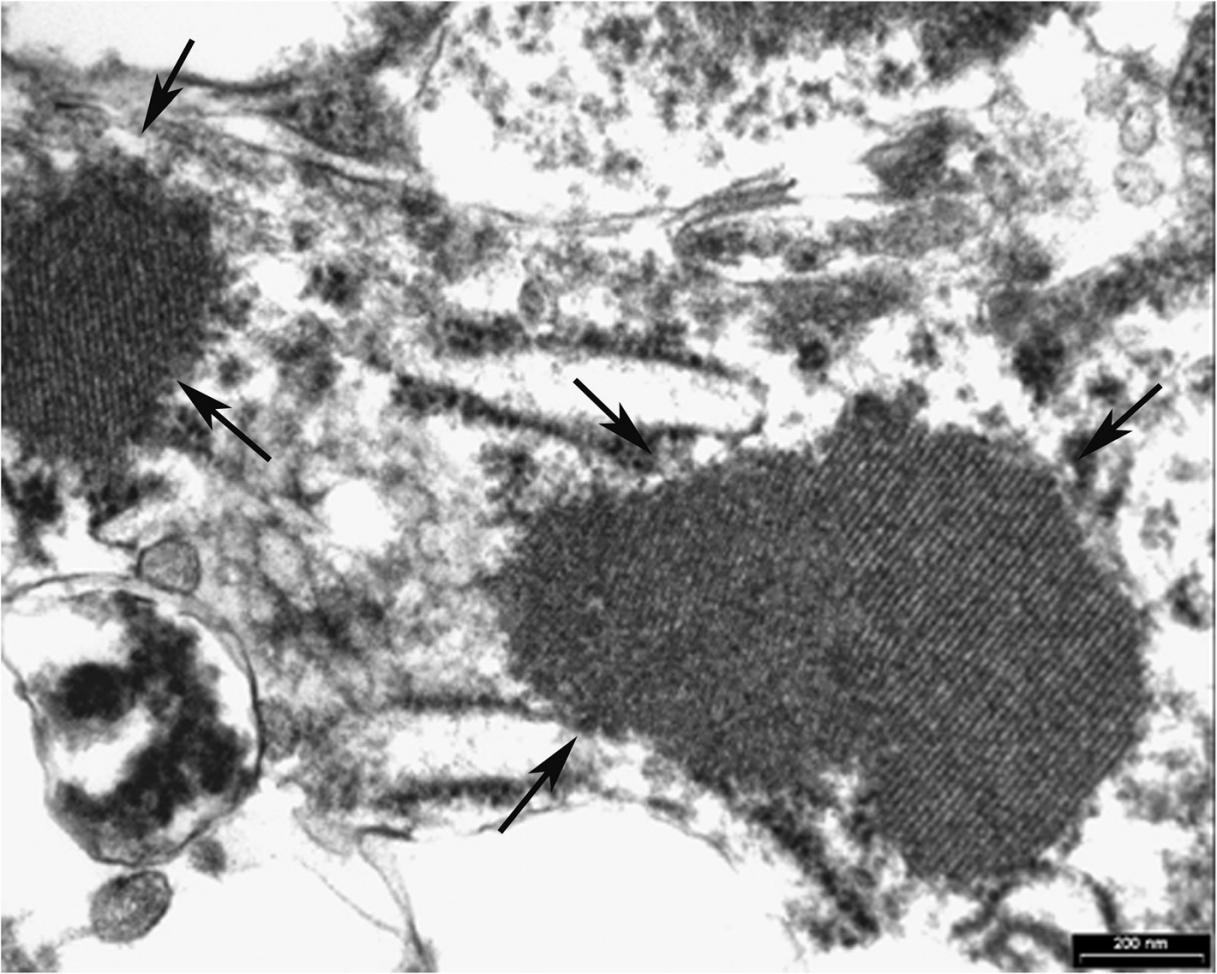
In an ultrastructural study, neoplastic cells had cytoplasmic crystalline inclusions (*arrows*) showing unidirectional periodicity

On immunohistochemical analysis, the neoplasm showed focal immunoreactivity to muscle-specific actin, caldesmon and caldesmin. Ki 67 index was very low (Fig. 3a). Strong nuclear positivity to TFE3 was observed in all neoplastic cells (Fig. 3b). Immunostains for CD 34, CD10, microphthalmia transcription factor (MITF), myoglobin, S-100 protein, HMB-45, neuron-specific enolase, synaptophysin, chromogranin, cytokeratin, epithelial membrane antigen (EMA), cyclin D1 and alpha-inhibin were negative. Electron microscopy (EM) examination revealed cytoplasmic crystal inclusions showing periodic pattern of about 10 nm in channels resembling dilated sacs of endoplasmic reticulum (Fig. 4).

Because of immunohistochemical and ultrastructural features, such as focal immunoreactivity to muscle-specific markers (actin, desmin, caldesmin), negativity to other makers, strong and diffuse nuclear positivity to TFE3 and the presence of cytoplasmic crystal inclusions, the final pathologic diagnosis was primary uterine alveolar soft part sarcoma of uterine corpus. No images suggestive of other primary or metastatic lesions were observed on abdominal ultrasound, chest X-ray, total computed tomography or bone scan.

Seven months after surgery, the patient was free of disease. In fact, when she was readmitted for further examination, no abnormalities were found in both physical examination and imaging studies.

### Discussion

ASPS is a rare malignant neoplasm, accounting for 0.5–1 % of all soft part sarcomas [33]. ASPS that originate from the uterine corpus is even more rare, with only 10 previous cases reported in the English literature [4, 5, 27–32]. The main clinical and morphological features of 11 cases (including our present case) are summarized in Tables 1 and 2.

**Table 1:** R1: clinical features of ASPS of the uterus corpus

Authors and years	Age	Clinical symptoms	Therapy	Follow-up
Gray et al. (1986) [27]	43	Metromenorrhagia	Total hysterectomy	NED 9 Mo
Nolan et al. (1990) [28]	14	Menorrhagia and expulsion of a necrotic mass per vagina	HBSO	NED 80 Mo
Guillou et al. (1991) [4]	40	Abdominal pelvic pain, intermenstrual bleeding	Total hysterectomy	NED 48 Mo
Burch et al. (1994) [29]	47	Intermenstrual bleeding	HBSO	NED12 Mo
	37	Hypermenorrhea	Total hysterectomy	NED 66 Mo
Nielsen et al. (1995) [30]	30	Menometrorrhagia	Total hysterectomy	NED 66 Mo
Radig et al. (1998) [31]	50	Abnormal uterine bleeding	HBSO	NED 84 Mo
	36	Intermenstrual spotting, dysmenorrhoea	Hysterectomy	NED 8 Mo
Kasashima et al. (2007) [5]	50	Abnormal uterine bleeding	HBSO, lymph	NED 38 Mo
Zhang et al. (2012) [32]	57	Abnormal uterine bleeding	HBSO, lymph chem	NED 1 Mo
Present case	66	Abnormal uterine bleeding	HBSO	NED 7 Mo

*ASPS* alveolar soft part sarcoma, *HBSO* hysterectomy with bilateral salpingo-oophorectomy, *NED* not evidence of disease, *Mo* months

**Table 2:** Pathological findings of ASPS of the uterus corpus

Authors and years	Macroscopic findings	Microscopic findings	Immunohistochemical findings	Electron microscopy
Gray et al. (1986) [27]	Uncapsulated, circumscribed intramyometrial nodule, 0.4 cm	Large cell, arranged in organoid appearance separated by delicate fibrovascular stroma	ND	Membrane bound crystalline granule inclusions in the cytoplasm
Nolan and Gaffney (1990) [28]	Mass of 7 cm, bulging into endometrial cavity	Large cells with granular and vacuolated cytoplasms, organoid arrangement, separated by fibrovascular septae. PASDR crystals	ND	Intracytoplasmic crystalline inclusions
Guillou et al. (1991) [4]	3 × 2.5 × 2.5 cm well-circumscribed intramyometrial nodule	Uncapsulated lesion pushing border delineated by endometrium, large cells with organoid arrangement, separated by fibrovascular septae, granular cytoplasms with crystals. Areas with nuclear pleomorphism	POS Vim, focal POS HMB45, NKI/C3, patchy dot-like cytokeratin lw POS	ND
Burch et al. (1994) [29]	3-cm endometrial polyp	Large cells with granular cytoplasm, crystals on PAS D	ND	Intracytoplasmic crystalline inclusions
Nielsen et al. (1995) [30]	1-cm intramural nodule	Large cell, arranged in organoid appearance separated by delicate fibrovascular stroma crystals on PAS D	ND	ND
	3.5-cm submucosal nodule	Large cell, arranged in organoid appearance separated by delicate fibrovascular stroma, crystals on PAS D	ND	ND
Radig K et al. (1998) [31]	3-cm well-circumscribed yellow-whitish, intramural nodule	Pseudoalveolar and trabecular pattern, polygonal and round cells vesicular nuclei, with nucleoli, crystals on PAS D	NEG for S100, Ck, EMA, POS caldes	Intracytoplasmic crystalline inclusions
	4.5-cm well-circumscribed, intramural nodule	Pseudoalveolar pattern, granular	ND	Intracytoplasmic crystalline inclusions absent
Kasashima S et al. (2007) [5]	1.9 × 1.9 × 1.0 cm greyish, endometrial exophytic nodule	Large cells with organoid arrangement, separated by fibrovascular septae, granular cytoplasms with crystals	Nuclear POS: TFE3, PGR, ER, CD10	ND
		Vesicular nuclei and pleomorphic nuclei and multinucleated cells. Mitoses very rare. No M Lym	NEG: Vim, caldes, Sma, Myo, HMB45, S100, EMA, Chrom, Syn, Myoge, Myogl	
Zhang et al. (2012) [32]	2.4 × 2.0 × 1.8 cm yellow-whitish exophytic tumour, sub-endometrium of the lower uterine segment	Large cells with organoid arrangement, separated by delicate fibrovascular septae, granular cytoplasms with crystals M lymph	NEG: Ae1/AE3, EMA, Sma, Des, S-100, CD10, Synapt, Chrom	ND
			Ki67: 5 % diffuse POS Nuclear TFE3	
Present case	5-cm intramural nodule, well-circumscribed, with irregular border , soft yellow, brownish and grey, with a large haemorrhagic zone centrally located	Solid “non-organoid” pattern, abundant eosinophilic granular cytoplasm, with distinct border, and vesicular nuclei and prominent nucleolus	Focal POS to Sma, desm, Caldes and diffuse and strong nuclear POS to TFE3 NEG: Ae1/AE3, EMA, Sma, Des, S-100, CD10, NSE, Synapt, Chrom, inh, MiTF	Intracytoplasmic crystalline inclusions
		Areas with spindle elements with nuclear, pleomorphism, hyperchromasia, nuclear cytoplasmic pseudo-inclusions and multinucleations	Diffuse POS nuclear TFE3	

*ASPS* alveolar soft part sarcoma, *Caldes* caldesmin, *Chrom* chromogranin, *CK* cytokeratin, *EMA* epithelial membrane antigen, *ER* oestrogen receptor, *caldes* caldesmon, *Immu* immunohistochemistry, *inh* alpha-inhibin, *Lym* lymph node, *lw* low weight, *MLymph* lymphonodal metastasis, *MITF* microphthalmia transcription factor, *Mo* months, *Myoge* myogenin, *Myogl* myoglobin, *ND* not done, *NSE* neuron-specific enolase, *PASDR* periodic acid-Schiff diastase resistant, *PGR* progesterone receptor, *POS* positivity, *Sma* muscle-specific actin, *Synapt* synaptophysin, *TFE3* transcription factor 3, *Vim* vimentin

The age at diagnosis ranged from 14 to 66 years (median, 43 years). Abnormal uterine bleeding was present in all patients. The size of the neoplasm varied from 0.4 to 7 cm (median, 3 cm) in diameter. In the majority of cases, the neoplasm was an intramural nodule. The diagnosis of ASPS in all cases was supported by histologic and EM examination, which revealed an alveolar pattern and the presence of cytoplasmic crystalline inclusions (Table 2).

Usually, the tumour nuclei were round-to-polygonal and vesicular, with prominent nucleoli, but cells with marked variation in nuclear size, nuclear cytoplasmatic inclusions and multinucleation, with very few mitoses have been observed in our case and in other ASPSs in the uterine corpus [4, 5] and extragenital sites [3].

In our case, the alveolar features were completely lost and the tumour show a solid, non-alveolar pattern and the nuclei have marked variation in nuclear size, nuclear-cytoplasmatic inclusions and multinucleation. The correct pathological diagnosis has been made by immunohistochemical and ultrastructural features, which revealed focal immunoreactivity to muscle-specific markers (actin, desmin, caldesmon), negativity to other makers, strong and diffuse nuclear positivity to TFE3 and the presence of cytoplasmic crystal inclusions.

Moreover, the lesion was considered primary uterine alveolar soft part sarcoma because no images suggestive of other primary or metastatic lesions were observed on abdominal ultrasound, chest X-ray, total computed tomography or bone scan.

On the contrary, the review of literature revealed an immunohistochemical profile not very consistent with variable staining using other markers such as vimentin, desmin, myoglobin, muscle-specific actin, S-100 protein, HMB-45, neuron-specific enolase, synaptophysin, chromogranin, cytokeratin, EMA and NK1-C3 (melanoma-specific antibody) (Table 2). Thus, in our case, nuclear over-expression of TFE3 can be considered as an essential diagnostic marker for a correct pathological diagnosis. It has been demonstrated that aberrant and strong nuclear expression of TFE3 is seen exclusively in tumours which contain the TFE3 gene fusions [11], such as ASPS and rare paediatric renal carcinomas [12]. TFE3 immunoreactivity was first tested in the female genital tract in a case of ASPS located in the uterine cervix by Roma et al. in 2005 [26]. TFE3 immunoreactivity in cases of uterine corpus of ASPSs was evaluated only in our case and in the examples reported by Kasashima et al. and by Zhang L et al. [5, 32] (Table 2). The presence of crystalline inclusions on electron microscopy further supports the diagnosis of uterine ASPS.

ASPS of the uterine corpus enters in the differential diagnosis with other neoplasms presenting an admixture of spindle-to-epithelioid cells with eosinophilic-to-clear cytoplasm such as epithelioid smooth muscle tumours, epithelioid endometrial stromal tumours, perivascular epithelioid cell tumours (PECOMAs), uterine rhabdoid tumours, carcinomas, melanoma and malignant paraganglioma. These tumours characteristically do not show expression of TFE3 on immunohistochemical analysis and have other peculiar morphologic and immunohistochemical features.

Epithelioid smooth muscle tumours are composed by mixtures of epithelioid, clear cell. A transition to typical smooth muscle cells in most instances confirms the smooth muscle nature of these tumours [34] and are diffuse immunoreactive for actin and desmin.

Endometrial stromal tumours with a prominent component of epithelioid cells and abundant eosinophilic cytoplasm usually show areas with fusiform cells, characteristic arterioles and strong diffuse cytoplasmic immunoreactivity for vimentin and for CD10 [35].

PECOMAs can arise in the uterus and are characterized by varying amounts of spindle and epithelioid cells with clear to eosinophilic cytoplasm, with immunoreactivity for melanocytic markers, most frequently HMB-45 [36].

Malignant rhabdoid tumour is a highly aggressive tumour in adults and rapidly fatal and was first reported to have been found in the uterus in 1989 [37]. Histologically, this rare neoplasm shows solid sheets of large cells with deep eosinophilic cytoplasm, eosinophilic hyaline cytoplasmic inclusions, eccentric vesicular nuclei and prominent nucleoli. On immunohistochemical analysis, neoplastic cells reveal cytoplasmic staining for vimentin [38] and keratins [38, 39].

Melanoma and carcinoma can be differentiated from ASPS because of greater cytologic atypia, pleomorphism, higher mitotic activity and immunoreactivity, respectively, for epithelial makers and for melanoma-specific antibodies.

Malignant paraganglioma is characterized by polygonal to oval cells arranged in distinctive cell balls, called Zellballen, and shows more pronounced cell pleomorphism and immunoreactivity for neuroendocrine markers, such as neuron-specific enolase, protein gene product 9.5, synaptophysin and the presence of neurosecretory granules on EM examination [40].

In extragenital sites, ASPS presents an indolent clinical course with unpredictable prognosis. Metastases to the lungs, bone and brain are the main cause of death [41, 42]. Lesions with size less than 5 cm in diameter seem to be correlated with a more favourable outcome [43]. ASPS in the uterine corpus has a better prognosis than ASPSs in the soft tissues and other cases located in the vagina. All patients with ASPS located in the uterine corpus were alive and well at the time of the last follow-up (Table 1). In contrast, one of six patients with vaginal ASPS died as a result of the tumour. Initially, this patient had a recurrence 4 months after local excision and external radiation therapy and then died of the disease with pulmonary metastases 25 months later [23, 24]. Another patient with vaginal ASPS had a recurrent 1.5-cm mass, 4 months after the initial diagnosis [25].

The more favourable prognosis in ASPS located in the uterine corpus may be due to small tumour size, anatomical location or relative short duration of follow-up. Indeed, in all patients, the ASPS of the uterine corpus, except for the present case and the case reported by Nolan and Gaffney, measured less than 5 cm [28] (Table 2). Only in the case reported by Zhang et al. was the pelvic and para-aortic lymph node metastasis observed at diagnosis, but the duration of follow-up in this case was short (9 months) and it is not possible to establish its outcome [32].

## Conclusions

An essential diagnostic marker in this unusual presentation of neoplasm is the nuclear over-expression of TFE3 as well as ultrastructural study, which reveals the presence of peculiar cytoplasmic crystalline inclusions.

Moreover, in our opinion, a larger number of cases of ASPS in the female genital tract with longer follow-up and pathological findings including sizes should help to better define the biological nature of ASPS in the uterine corpus. In addition, although lymph nodes metastasis was observed only in the case of Zhang et al., surgical staging with complete pelvic lymph nodes sampling could be useful to evaluate therapy and prognosis of this rare neoplasm.

## Consent

Written informed consent was obtained from the patient for publication of this case report and any accompanying images.

## Abbreviations

ASPS: alveolar soft part sarcoma
Caldes: caldesmon
Chrom: chromogranin
CK: cytokeratin
EM: electron microscopy
EMA: epithelial membrane antigen
ER: oestrogen receptor
Desm: desmin
HBSO: hysterectomy with bilateral salpingo-oophorectomy
Immu: immunohistochemistry
inh: alpha-inhibin
Lym: lymph node
lw: low weight
MLymph: lymphonodal metastasis
MITF: microphthalmia transcription factor
Mo: months
Myoge: myogenin
Myogl: myoglobin
NED: not evidence of disease
ND: not done
NSE: neuron-specific enolase
PASDR: periodic acid-Schiff diastase resistant
PGR: progesterone receptor
POS: positivity
Sma: muscle-specific actin
Synapt: synaptophysin
TFE3: transcription factor 3
Vim: vimentin
